# NetBenchmark: a bioconductor package for reproducible benchmarks of gene regulatory network inference

**DOI:** 10.1186/s12859-015-0728-4

**Published:** 2015-09-29

**Authors:** Pau Bellot, Catharina Olsen, Philippe Salembier, Albert Oliveras-Vergés, Patrick E. Meyer

**Affiliations:** 1 Universitat Politecnica de Catalunya BarcelonaTECH, Department of Signal Theory and Communications, UPC-Campus Nord, C/ Jordi Girona, 1-3, Barcelona, 08034 Spain; 2 Bioinformatics and Systems Biology (BioSys), Faculty of Sciences, Université de Liège (ULg), 27 Blvd du Rectorat, Liège, 4000 Belgium; 3 Machine Learning Group, Université Libre de Bruxelles, Brussels, Belgium; 4 Interuniversity Institute of Bioinformatics Brussels, (IB)², Brussels, Belgium

**Keywords:** Bioconductor package, Gene regulatory networks, Gene expression, Gene regulation network reconstruction, Synthetic genetic networks, Benchmark

## Abstract

**Background:**

In the last decade, a great number of methods for reconstructing gene regulatory networks from expression data have been proposed. However, very few tools and datasets allow to evaluate accurately and reproducibly those methods. Hence, we propose here a new tool, able to perform a systematic, yet fully reproducible, evaluation of transcriptional network inference methods.

**Results:**

Our open-source and freely available Bioconductor package aggregates a large set of tools to assess the robustness of network inference algorithms against different simulators, topologies, sample sizes and noise intensities.

**Conclusions:**

The benchmarking framework that uses various datasets highlights the specialization of some methods toward network types and data. As a result, it is possible to identify the techniques that have broad overall performances.

**Electronic supplementary material:**

The online version of this article (doi:10.1186/s12859-015-0728-4) contains supplementary material, which is available to authorized users.

## Background

Despite extensive knowledge of individual genes, we are still far from understanding the regulation mechanisms happening inside biological cells. In order to gain a system-level understanding, it is necessary to examine how genes interact on a large-scale level.

Some specific genes called transcription factors (TF) bind to the promoter regions of target genes (TG) and can activate or inhibit a TG’s expression. Therefore, genes do not work in isolation; they are connected in highly structured networks. Gene Regulatory Networks (GRNs) represent this set of relationships.

Reconstructing gene regulatory networks from expression data is a very difficult problem that has seen a continuously rising interest in the past decade, and presumably this trend will continue in the years to come due to the rich set of applications in biotechnological fields (biofuel, food, etc.) as well as in the biomedical field (drug design, cancer signatures, etc.). Several papers have compared and evaluated different network reconstruction methods [1–5]. However, a free open-source tool providing a fully reproducible benchmark is yet missing. Furthermore, in each state-of-the-art study, only one synthetic data generator has been used: either the GeneNetWeaver (GNW) simulator [3] in [4] and [5] or the SynTReN simulator [1] in [2]. As a result, different conclusions about the best performing methods have been obtained in each study. Finally, most reviews do not evaluate the changes of performances of the methods as a function of the number of genes, of the number of experiments or of the intensity of noise for multiple simulators and topologies (SynTReN, GNW, E.coli, S. cerevisae, etc.).

Hence, we propose a new extensive benchmarking framework that is fully reproducible with just one line of code and can also be easily modified to change the experimental setting or introduce a new inference algorithm. Our benchmarking strategy clearly shows that some methods perform very well on one of these artificial generators but can have poor results on another. This strongly suggests the importance of a tool that is able to test, both simply and broadly, any new proposed method. Some reviews such as [6] and [7] evaluate the behavior of different GRN reconstruction methods in real data corresponding to well known microbes in [6] and to ovarian cancer cells in [7]. Although real data represents a theoretically more interesting challenge than artificial data, they suffer from several drawbacks. First, the different algorithms are tested based on only partial knowledge of the underlying network [8], where a false positive could be a still undiscovered true positive. Second, the intensity of noise is uncontrollable. Hence, assessing a method’s robustness to varying intensities of noise cannot be done easily with real data. However, different noise intensities and distributions are observed from different measurement platforms (i.e. microarray vs RNAseq) as well as from different organisms. As a result, assessing the performance of any reverse-engineering algorithm on a few real datasets gives little information on its performance on other type of organisms and measurement platforms.

For this reason, we provide a Bioconductor package that, by default, compares 10 variations of 5 datasets having more than 100 expression-measurements each. In other words, the package compares methods on 50 datasets, each with very different samples and even different amounts of noise. Using realistic artificial data allows for large number of samples that in turn, allows for reliable statistical measures indicative of performances and robustness. So far, no consortium nor database focusing on real data has assembled several thousands of homogeneous expression samples (coming from the same experimental platform), that would allow for a similar benchmark. In this paper, we argue that a first step to support a new network inference method is to demonstrate its ability to recover regulatory networks from a broad set of realistic artificial datasets, where the truth is known and where the noise is controlled. Then, of course, a second step would be the analysis of the algorithms on real data (for example, coming from model organisms).

In this study we will show that our benchmarking strategy is highly informative for evaluating the performance and robustness of network reconstruction methods. Indeed, in this paper, we evaluate more than ten state-of-the-art reconstruction techniques using more than 50 datasets from different simulators in a high number of genes and low number of experiments scenario.

With this study we found that no single method is the best across different sources of data, but at the same time this study also shows that some techniques, such as CLR [9], are rather good in average. We also tested the sensitivity of these methods with regard to different kinds of noise and to the number of experiments. Those experiments highlight which methods are more adapted to the common scenario (i.e. few samples and high noise). Although often overlooked, reproducibility is an important issue in the field of benchmarking. Hence, in order to provide the scientific community with tools allowing the full reproduction of the tests as well as their extension or modification, we provide our benchmarking tools in a Bioconductor package. Table 1 summarizes the most important aspects concerning benchmarking and compares the features included in previously published reviews and the one described here.

**Table 1:** Reviews of GRN reconstruction methods and their characteristics

Review	[2]	[4]	[5]	This study
Number of variables	100	∈ [1643,5950]	∈ [10,100]	∈ [300,2000]
Topologies	Yeast	E. coli & S. cerevisiae & S. aureus	Yeast & E. coli	Synthetic & Yeast & E. coli
Number of methods compared	4	37	29	10
Simulators	SynTReN	GNW	GNW	Rogers & GNW & SynTReN
Number of experiments	∈ [20,200]	∈ [160,805]	∈ [10,100]	∼150
Impact of number of experiments	–	–	$\checkmark $	$\checkmark $
Impact of noise	–	–	–	$\checkmark $
Dataset availability	–	$\checkmark $	–	$\checkmark $
Benchmark extension	–	$\checkmark $	–	$\checkmark $
Possibility to change parameters	–	–	–	$\checkmark $

## Materials and methods

### Benchmarking process

**Fig. 1:**
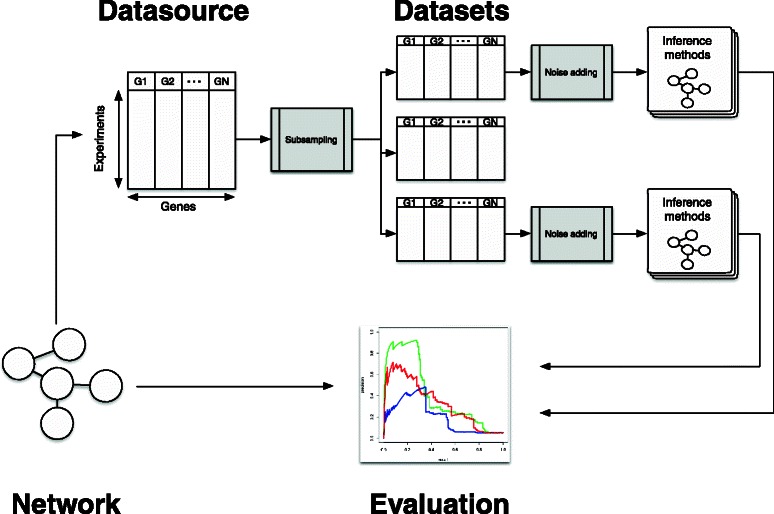
Workflow of the network evaluation process

In order to provide a sound and fair comparison of the different methods, the use of various simulators is essential. A large set of gene expressions generated by various simulators is collected in what we call “Datasource” (see Fig. 1).

At this stage, the data generated by the simulators is free of noise. The noise will be added later so that it is possible to control its properties independently of the simulators and also to provide fully reproducible tests. This study involves data generated by three different GRN simulators:

**GNW** The GNW simulator [3] generates network structures by extracting parts of known real GRN structures capturing several of their important structural properties. To produce gene expression data, the simulator relies on a system of non-linear ordinary differential equations (ODEs).

**SynTReN** The SynTReN simulator [1] generates the underlying networks by selecting sub-networks from *E. coli* and *Yeast* organisms. Then the experiments are obtained by simulating equations based on Michaelis-Menten and Hill kinetics under different conditions.

**Rogers** The data generator described in [10] that will be referred as *Rogers* (as in [11]) relies on a power-law distribution on the number of connections of the genes to generate the underlying network. The steady state of the system is obtained by integrating a system of differential equations simulating only knockout data.

**Data generation process** Using these simulators, five large datasources involving many noise-free experiments have been generated.

The characteristics of these datasources are detailed in Table 2. In order to generate these datasources we have simulated multifactorial data with SynTReN and GNW, which provides less information than than extensive knockout, knockdown or time series experiments [12]. However, multifactorial data are the most common type of expression datasets because of experimental constraints.

**Table 2:** Datasources used in this study and their characteristics

Datasource	Name	Topology	Experiments	Genes	Edges
*R* *o* *g* *e* *r* *s*_1000_	R1	Power-law	1000	1000	1350
		tail topology			
*S* *y* *n* *T* *R* *e* *N*_300_	S1	E. coli	800	300	468
*S* *y* *n* *T* *R* *e* *N*_1000_	S2	E. coli	1000	1000	4695
*G* *N* *W*_1565_	G1	E. coli	1565	1565	7264
*G* *N* *W*_2000_	G2	Yeast	2000	2000	10392

The next step of the benchmarking process is to randomly subsample those datasources in order to generate a large set of different but homogeneous datasets. Each dataset has a different number of experiments extracted from one of the five datasources. In the design we prevent the same experiment to be used several times in the same dataset, but it can appear in different datasets (it is worth noting that because of the high number of samples provided in the datasource, the probability of many identical samples in several datasets is very low in all our tested setups). Each dataset is then contaminated with noise with a slightly different signal-to-noise ratio; this aims to reproduce the variability in the real microarray generation process within the same laboratory or between different ones. In the present study, we have chosen to add a mixture of Gaussian noise and lognormal noise to resemble to characteristics of the experimental noise observed in microarrays [13]. The first noise, called “local” noise is an additive Gaussian noise with zero mean and a standard deviation (*σ*_*Local*(*g*)_) that is around a percentage (*κ* *%*) of the gene standard deviation (*σ*_*g*_). Therefore, the Signal-to-Noise-Ratio (SNR) of each gene is similar. The local noise standard deviation can be formulated as follows:

(1) $$ \sigma_{Local(g);\kappa\%}=\sigma_{g} \frac{ \mathcal{U}(0.8\kappa,1.2\kappa)}{100},  $$

where $\mathcal {U}(a,b)$ denotes the uniform distribution between *a* and *b*. This kind of noise will be referred to as local noise.

Additionally, we add an independent lognormal noise called “global” noise in the sequel. The standard deviation of this noise (*σ*_*Global*_) is the same for the whole dataset and is a percentage (*κ*_*g*_ *%*) of the mean variance of all the genes in the dataset ($\overline {\sigma _{g}}$). It is defined as follows:

(2) $$ \sigma_{Global;\kappa_{g}\%}=\overline{\sigma_{g}} \frac{ \mathcal{U}(0.8\kappa_{g},1.2\kappa_{g})}{100}.  $$

We have chosen to add a range of 40 % around *κ* and *κ*_*g*_ in order to add some variability to the different generated datasets. This range allows the various datasets to have some heterogeneity in noise but ensures at the same time that they are not too different from the originally specified values *κ* and *κ*_*g*_. We have chosen this value to reflect our experience with real data. Nevertheless, in addition to this range (40 %), we also tested bigger and smaller ranges (60 %, 20 % and 10 %) around *κ* and *κ*_*g*_, and the conclusions reached by the benchmark are equivalent. In Fig. 1, a flowchart illustrates the process. In our implementation, the various datasources have previously been generated with the in silico simulators and stored. As a result, the process is fast as no ODEs have to be computed. Moreover, this makes the reproducibility of the tests much easier, as it is not necessary to interact and parametrize the various simulators (with some of them being quite complex). Although no artificial generator is really equivalent to real data, an in silico analysis gives reliable guidelines on algorithms’ performance in line with the results obtained on real data sets [14]. Additionally, the use of several different datasources coming from different simulators renders the subsequent analysis of methods more credible before any use on real data.

**Implementation in NetBenchmark package** The different datasets are automatically loaded with the package, and are listed in character vector named Availabledata, which contains the names of the datasources. For each of these, we provide the simulated data and the underlying network. The former is a data.frame containing a simulated microarray experiment, where each row contains an experiment and each column contains the gene expression. The true underlying network is in the form of an adjacency matrix.

The dataset generation process is implemented in the function datasource.subsample, that returns a list with datasets.num number of elements. Each element of the list contains a data.frame of the subsampled datasource with the same number of genes and different numbers of experiments. The user could also specify the number of experiments. Moreover, the amount of local noise and global noise are controlled by parameters local.noise and global.noise, respectively. The distribution of noise with the variable noiseType that can be (“normal” or “lognormal”).

### Evaluation protocol

A network reconstruction problem can be seen as a binary decision problem. After thresholding the edge list provided by the GRN algorithm, the final decision can be seen as a classification. For each possible pair of nodes, the algorithm either infers an edge or not. As a result, we get correct connections and misclassified connections. Therefore, the performance evaluation can be done with the usual metrics of machine learning like Receiver Operating Characteristic (ROC) and Precision and Recall (PR) curves. ROC curves display the relative frequencies of true positives to false negatives for every predicted link of the edge list. Whereas the PR curves shows the relative precision (the fraction of correct predictions) versus recall (also called sensitivity) that is equivalent to the true positive ratio. These relative frequencies are also computed for every link. For a discussion of the relation between PR and ROC curves, we refer the reader to [15].

Note that since the provided expression datasets do not contain temporal information, predicting self-interactions is irrelevant. Moreover, most of the state-of-the-art methods do not attempt to recover this kind of relationships. So, we do not consider self-interactions to compute those evaluation metrics.

The DREAM5 challenge [4] and its previous editions [12] have established a de-facto protocol to evaluate an inferred network. The protocol consists in computing the PR or ROC curves, and in measuring the Area Under the Precision Recall curve (AUPR) or Area Under ROC curve (AUROC). This approach gives an estimation of the global behavior of the method. However, other papers have evaluated the inferred networks using only the most reliable inferred connections [8, 16].

We have adopted the latter approach, evaluating the inferred networks using only the top best *x* % of the total number of possible connections (if the network has *G* genes, then the total number of possible connections is *G* ^2^−*G*). This leads to a total of *t* evaluated connections that will be different for each datasource.

We use as performance measures the mean precision, the AUPR and the AUROC in the top best *t* inferred connections. These measures could be obtained from a directed or undirected evaluation. The former evaluates the existence of an edge and its direction while the latter only evaluates the existence of an edge.

**Implementation in NetBenchmark package** The evaluation is performed by the function evaluate(inf.net,true.net,sym) which compares the inferred network (inf.net) with the true underlying network (true.net). It returns the resulting confusion matrices for each threshold value. This could be obtained from a directed or undirected evaluation (specified with the logical argument sym).

**GRN inference methods** In this section, we provide a brief overview of the different GRN Inference approaches: algorithms based on co-expression, information-theoretic approaches, and feature selection approaches.

We use the following notation: *X*_*i*_ denotes the expression levels of the *i*th gene in every experiment. It is a vector with *N* observations corresponding to the various experiments. Finally, the particular gene expression level of the *k*th experiment of the *i*th gene is denoted by *x*_*ik*_.

**1) Co-expression algorithms** These methods assume that similar patterns in gene expression profiles under different conditions are evidence of relationships between genes. Since the coordinated co-expression of genes encodes interacting proteins, studying co-expression patterns can provide insight into the underlying cellular processes.

Co-expression algorithms reconstruct a network by computing a similarity score for each pair of genes. The most simple co-expression method uses the correlation between genes as similarity measure. If the correlation is greater than a threshold, then the genes are connected in the graph in an undirected way (because the correlation is symmetric).

But, in practice these methods are not used for transcriptional network reconstruction because they recover indirect regulatory relationships. For example, if gene *A* regulates gene *B* and this last one regulates gene *C*. Co-expression algorithms will find a relationship between gene *A* and gene *C* even though it is an indirect effect. To avoid the inclusion of these indirect effects in the recovered network, a post-processing step should be carried on.

**GeneNet** In [17], the authors propose a heuristic for statistically learning a causal network. It relies on the conversion of a network inferred through correlation into a partial correlation graph. Then, a partial ordering of the nodes is assigned by means of a multiple testing of the log-ratio of standardized partial variances. This allows identifying a directed acyclic causal network as a sub-graph of the partial correlation network.

**MutRank** MutRank [18] ranks the correlation between every pair of genes and this rank is taken as the score that describes the similarity between genes. For every gene *i*, the Pearson’s correlation (corr) with all other genes *l* is computed and ranked:

(3) $$ r_{ij} = \operatorname*{\,rank}_{j} (\operatorname*{\,corr} (X_{i},X_{l}),\forall i \ne l).  $$

As this expression is not symmetric, the final confidence score assigned between genes *i* and *j* is computed as the geometric mean of the scores obtained between gene *i* and *j* and vice versa:

(4) $$ s_{ij}=\frac{r_{ij}\cdot r_{ji}}{2}.  $$

**Zscore** Zscore [19] is a method that assumes interventional data, more concretely knockout experiments that lead to a change in other genes. The assumption is that the knocked-out gene *i* in the experiment *k* affects more strongly the genes that it regulates than the others. The effect of the gene *i* over gene *j* is captured with the Zscore *z*_*ij*_:

(5) $$  z_{ij}=\left|\frac{x_{jk}-\mu_{X_{j}}}{\sigma_{X_{j}}}\right|,  $$

assuming that the *k*th experiment is a knockout of gene *i*, $\mu _{X_{j}}$ and $\sigma _{X_{j}}$ are respectively the mean and standard deviation of the empirical distribution of the gene *j*. To apply the original method, one needs to know which knockouts are done in each experiment. However, in practice, one can assume that the knocked-out gene is the one corresponding to the minimum value in the experiment *k*: arg min_*l*_(*x*_*lk*_)=*i*. With this generalization, the method can be applied to any type of data like multifactorial or knockdown data. If the same gene is detected to be knocked-out in various experiments, then the final Zscore is the mean of the individual Zscore values.

**2) Information-theoretic approaches** These approaches use a generalization of the pairwise correlation coefficient that is called mutual information (*M*_*ij*_) [20]. It measures the degree of dependence between two genes *X*_*i*_ and *X*_*j*_.

(6) $$ M_{ij}=\sum_{X_{i}}\sum_{X_{j}} p(X_{i},X_{j}) \log_{2} \frac{p(X_{i},X_{j})}{p(X_{i})p(X_{j})},  $$

where *p*(*X*_*i*_,*X*_*j*_) is the joint probability distribution function of *X*_*i*_ and *X*_*j*_, and *p*(*X*_*i*_) and *p*(*X*_*j*_) are the marginal probability distribution functions of *X*_*i*_ and *X*_*j*_ respectively [20].

**Relevance network** The RELNET [21] is the simplest method based on mutual information. For each pair of genes, the mutual information *M*_*ij*_ is estimated and the edge between genes *i* and *j* is created if the mutual information is above a threshold. Despite that mutual information is more general than the Pearson correlation coefficient, in practice thresholding the *M*_*ij*_ or Pearson correlation produces similar results [22].

**CLR** The Context Likelihood or Relatedness network (CLR) method [9] is an extension of the previous method. The method derives a score that is associated to the empirical distribution of the mutual information values. In practice, the score between gene *i* and gene *j* is defined as follows:

(7) $$ \begin{aligned} c_{ij} &=\sqrt{{c^{2}_{i}}+{c^{2}_{j}}}, \, \, \text{with} \, \, c_{i} =\max \left(0,\frac{M_{ij}-\mu_{M_{i}}}{\sigma_{M_{i}}} \right) \, \, \text{and} \, \,\\ c_{j} &=\max \left(0,\frac{M_{ji}-\mu_{M_{j}}}{\sigma_{M_{j}}} \right). \end{aligned}  $$

The mean and standard deviation of the empirical distribution of the mutual information between both genes are denoted by $\mu _{M_{i}}$ and $\sigma _{M_{i}}$, which are defined as:

(8) $$  \mu_{M_{i}} =\frac{1}{G} \sum_{l=1}^{G} M_{il},\quad \sigma_{M_{i}} =\sqrt{\frac{1}{G-1} \sum_{l=1}^{G} (M_{il}-\mu_{M_{i}})^{2}} \,\,.  $$

This process can be seen as a normalization of the mutual information [23].

**ARACNE** The motivation of the Algorithm for the Reconstruction of Accurate Cellular NEtworks (ARACNE) [24] is that many similar measures between variables may be the result of indirect effects. In order to avoid the indirect effect, the algorithm relies on the “Data Processing Inequality” (DPI) which removes the weakest edge, that is the one with the lowest mutual information, in every triplet of genes.

**PCIT** The Partial Correlation coefficient with Information Theory (PCIT) [25] algorithm combines the concept of partial correlation coefficient with information theory to identify significant gene-to-gene associations.

Similarly to ARACNE, PCIT extracts all possible interaction triangles and applies DPI to filter indirect connections, but instead of mutual information it uses first-order partial correlation as interaction weights. The partial correlation tries to eliminate the effect of a third gene *l* on the correlation of genes *i* and *j*.

**C3NET** The Conservative Causal Core NETwork (C3NET) [26] consists of two main steps. In the first step pairwise mutual information is computed. Then, non-significant connections are eliminated, according to a chosen significance level *α*, between gene pairs. But the main difference is the second step, where only the most significant edge for each gene is selected. This edge corresponds also to the highest mutual information value among the neighboring connections for each gene.

The consequence of the second step is that the highest possible number of connections that can be reconstructed by C3NET is equal to the number of genes under consideration. C3NET does not aim at reconstructing the entire network underlying gene regulation but mainly tries to recover the core structure.

**3) Feature selection approaches** A GRN reconstruction problem can also be seen as a feature selection problem. For every gene, the goal is to discover its true regulators among all other genes or candidate regulators. This approach can integrate knowledge about genes that are not TFs and therefore reduce the search space.

Typically, this approach only focuses on designing a significance score *s*(*i*,*j*) that leads to a good ranking of the candidate regulations, such that true regulations tend to be at the top of the list since an edge is assigned between *i* and *j* if the evidence *s*(*i*,*j*) is larger than a threshold.

With the feature selection approach, the scores *s*(*i*,*j*) for all the genes are jointly estimated with a method that is able to capture the fact that a large score for a link (*i*,*j*) is not needed if the apparent relationship between *i* and *j* is already explained by another and more likely regulation.

**MRNET** The Minimum Redundancy NETworks (MRNET) [27] method reconstructs a network using the feature selection technique known as Minimum Redundancy Maximum Relevance (MRMR) [28], which is based on a mutual information measure. In order to get a network, the algorithm performs a feature selection for each gene (*i*∈ [1,*G*]) on the set of remaining genes (*j*∈ [1,*G*]∖*i*).

The MRMR procedure returns a ranked list of features that maximize the mutual information with the target gene (maximum relevance) and, at the same time, such that the selected genes are mutually dissimilar (minimum redundancy). For every gene, the MRMR feature selection provides a score of potential connections where the higher scores should correspond to direct interactions. The indirect interactions should have a lower scores because they are redundant with the direct ones. Then, a threshold is computed as in the RELNET method.

The MRNET reconstructs a network using a forward selection strategy, which leads to subset selection that is strongly conditioned by the first selected variables. The Minimum Redundancy NETworks using Backward elimination (MRNETB), uses instead a backward selection strategy followed by a sequential replacement [29].

**Genie3** The GEne Network Inference with Ensemble of trees (Genie3) [30] algorithm uses the random forests [31] feature selection technique to solve a regression problem for each of the genes in the network. In each of the regression problems, the expression pattern of the target gene should be predicted from the expression patterns of all transcription factors.

The importance of each transcription factor in the prediction of the target gene is taken as an indication of an apparent regulatory edge. Then these candidate regulatory connections are aggregated over all genes to generate a ranking for the whole network.

**How to benchmark a method** These previously presented methods are implemented or imported with the package. We have developed a wrapper with the with the parameters recommended in the original publications of each method. The only exception is the Genie3, for which we reduced the number of trees from 1000 to 500 in order to limit the computation time required for this method. Table 3 shows the computation time in seconds needed by the various methods for each datasource. The names of the wrappers of the GRN inference algorithms that are currently available are listed in Table 4.

**Table 3:** Evaluation of the computational complexity. Mean CPU time in seconds of each reconstruction method on the different datasources in a 2 x Intel Xeon E5 2670 8C (2.6 GHz)

Datasource	ARACNE	C3NET	CLR	GeneNet	Genie3	MRNET	MutRank	MRNETB	PCIT	Zscore
R1	2.483	2.367	0.409	9.377	1310.486	7.200	0.638	11.195	11.352	0.086
S1	0.106	0.215	0.059	0.917	183.266	0.120	0.056	0.406	0.333	0.010
S2	1.775	1.904	0.349	9.504	950.648	7.101	0.585	10.907	10.898	0.091
G1	10.442	6.795	1.079	29.612	2839.319	31.385	1.865	46.255	47.106	0.260
G2	25.551	12.189	1.750	53.792	4115.408	60.143	3.431	100.375	103.085	0.418

**Table 4:** Included GRN algorithms. GRN algorithms included in the current version (1.0) of the netbenchmark Bioconductor package

GRN Algorithms	Wrapper function
ARACNE [24]	aracne.wrap
C3NET [26]	c3net.wrap
CLR [9]	clr.wrap
GeneNet [17]	genenet.wrap
Genie3 [30]	genie3.wrap
MutRank [18]	mutrank.wrap
MRNET/B [27, 29]	mrnet.wrap & mrnetb.wrap
PCIT [25]	pcit.wrap
Zscore [19]	zscore.wrap

The package allows the user to reproduce as well as to modify the experiments reported in this paper. However, an important additional functionality is that it also allows new methods to be evaluated. In the current version of the netbenchmark package (1.0), it is possible to evaluate new unsupervised network inference methods. The method should infer the network from steady-state expression data, and should be able to perform this task with a number of experiments much lower than the number of genes. The last requirement is that the provided method is and be able to infer networks with thousands of genes. In order to benchmark a new method, a new wrapper has to be defined: fun(data). This function receives a numeric data.frame with the gene expression data in the argument data where the columns contain the genes and the rows the experiments. The function should return a matrix which is the weighted adjacency matrix of the network inferred by the algorithm. In order to benchmark this method against all the other algorithms of the package the following procedure should be followed:



For more information on this topic, we refer the interested reader to the vignette of the package where an example is provided.

## Implementation

NetBenchmark is a Bioconductor [32] package. As a results, the code is written primarily in R [*]. However, time-critical functions are written in C++ for greater speed. The package imports several CRAN and Bioconductor packages. Most of those provide competitive network inference methods that are used in our benchmark. The pipeline starts with a set of noise-free datasources coming from different GRN simulators that have been pregenerated for this package. The datasources are stored in grndata package [**] and are loaded automatically as input. These datasources are subsampled and contaminated with noise in order to generate datasets with enough variability to provide an informative and thorough comparison of GRN inference methods. This benchmarking process is detailed throughout the subsequents sections of the paper. A helper vignette and a webpage (see “ Availability and requirements ”) are also provided in order to unlock the full set of functionalities of the package including the ability of adding new methods in the benchmark.

[*] R Core Team: R: A Language and Environment for Statistical Computing. R Foundation for Statistical Computing, Vienna, Austria (2015). R Foundation for Statistical Computing. https://www.R-project.org.

[**] Bellot, P., Olsen, C., Meyer, P.E.: grndata: Synthetic Expression Data for Gene Regulatory Network Inference. (2014). R package version 1.0.0.

## Results

In this section, we present the results of the benchmark with the presented methodology and obtained with version 1.0 of the package (see “ Availability and requirements ”). For each datasource of Table 2, we generate ten datasets with around 150 experiments. We aim to reproduce common real microarray datasets that are typically constituted of much less experiments than genes. As explained in section “Benchmarking process”, we add two different types of noise: local and global. We perform a benchmark of the methods listed in Table 4 adding local Gaussian noise around 20 % of the standard deviation (*σ*_*Local*(*g*);20 *%*_, see Eq. 1) and global lognormal noise around 10 % (*σ*_*Global*;10 *%*_, see Eq 2). Additionally to this benchmark, we also analyze the different algorithms according to two different aspects: the impact of the noise and the influence of the number of experiments included in the datasets.

Table 5 presents the Area Under Precision Recall curve obtained in an undirected evaluation on the top 20 % (*A* *U* *P* *R*_20 *%*_) of the total possible connections for each datasource. The table also gives the mean and variance across the 10 different datasets.

**Table 5:** Performances of the various GRN inference methods on the datasources. AUPR in the top 20 % of the possible connections with a undirected evaluation for each GRN inference method on the different datasources of the benchmark with a 20 % local Gaussian noise and 10 % of global lognormal noise. The best statistically significant results tested with a Wilcoxon test are highlighted for each datasource. Results obtained with current version (1.0) of the package and are updated online

Datasource		ARACNE	C3NET	CLR	GeneNet	Genie3	MRNET	MutRank	MRNETB	PCIT	Zscore	Random
R1	mean	0.004	0.002	0.005	0.140	0.024	0.005	0.042	0.005	**0.177**	0.140	<0.001
	*σ*(×10^−3^)	1.1	0.789	1.22	16	2.97	1.26	7.27	1.26	16.1	13.6	0.0265
S1	mean	0.039	0.032	0.139	0.062	0.134	0.109	0.063	0.118	0.060	0.028	0.001
	*σ*(×10^−3^)	8.02	7.92	1.98	8.25	3.51	9.45	2.25	5.83	1.44	13.8	0.211
S2	mean	0.006	0.006	0.042	0.013	0.036	0.021	0.021	0.021	0.01	0.003	<0.001
	*σ*(×10^−3^)	1.19	1.63	1.55	1.56	1	2.76	0.959	2.01	0.522	1.46	0.1
G1	mean	0.106	0.100	**0.139**	0.085	0.108	**0.134**	0.034	0.084	0.063	0.001	<0.001
	*σ*(×10^−3^)	7.46	7.58	7.83	2.91	6.66	9.48	2.26	3.27	2.69	0.15	0.0141
G2	mean	0.101	0.095	0.106	0.037	0.069	**0.126**	0.025	0.058	0.044	<0.001	<0.001
	*σ*(×10^−3^)	11.4	9.95	4.49	1.62	3.44	9.49	1.43	2.23	2.16	0.0917	0.0265

p < 0.05

In order to assess the statistical significance of the results, we perform a Wilcoxon Rank sum test with Bonferroni correction [33] on *A* *U* *P* *R*_20 *%*_ values for each datasource. Then, the best result is highlighted in bold if its metric is statistically different from the remaining values. Note that several results may be highlighted for the same datasource if they are not statistically different from each other.

**Fig. 2:**
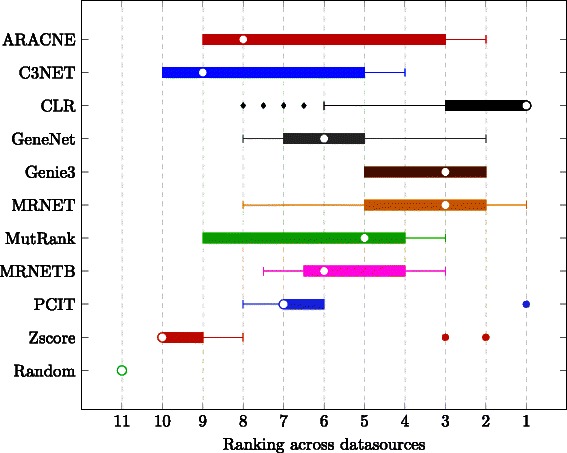
Boxplots of performance. Each box represents the statistics of a method with the ranking performance across all datasources, the smaller the rank the better. The white dot represents the median of the distribution, the box goes form the first to third quartile, while whiskers are lines drawn from the ends of the box to the maximum and minimum of the data excluding outliers that are represented with a mark outside the whiskers

In order to assess the overall behavior of each technique, we need to aggregate the different performances obtained on the different datasources. But as can be seen in Table 5, the *A* *U* *P* *R*_20 *%*_ values have different ranges for each datasource. Therefore, instead of aggregating *A* *U* *P* *R*_20 *%*_ values, we aggregate the rank of each method, the smaller the rank the better the algorithm. Figure 2 presents a boxplot of the rank of the different algorithms across all datasources. For more information on the boxplot, we refer the reader to [34].

Additionally, Table 3 shows the time needed by the various methods for each datasource (in seconds). This information allows to estimate the scalability of each method.

**Implementation in NetBenchmark package** In order to generate these results we use the main function netbenchmark. In listing ?? we present the different commands used in the netbenchmark function to generate the previously presented results, note that the random seed could be used to compare a new method on the same data than those used in the present study. Results are also available at online (see Project home page in section “Availability and requirements ”) where the results of the benchmark will be updated (with most recent version of the package) with new methods or updates of the presented algorithms.

### Noise sensitivity

**Fig. 3:**
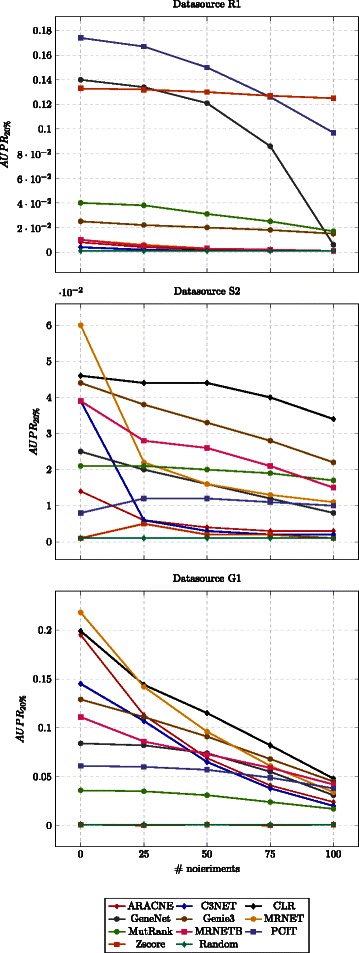
Plots of performance with different noise intensities. Each line represents a method (color coded), the mean performance over the ten runs is presented

Here we present a procedure in order to test the stability of the different algorithms in the presence of local Gaussian noise. To do so, we use all datasources in Table 2 increasing gradually the local noise intensity (increasing *κ* value of *σ*_*n*;*κ%*_), therefore decreasing the SNR. In this study we also use subsampled datasources of 150 experiments in order to derive the effect of noise on the various GRN reconstruction methods and being able to compare them with the results obtained at the previous study. In Table 6 we present the mean values of the AUPR in an undirected evaluation on the top 20 % of the total possible connections at each dataset. For each *σ*_*n*;*κ%*_ value, we perform ten different trials and the performance metrics (*A* *U* *P* *R*_20 *%*_) are the average of the different trials. In Fig. 3 the results of the datasources that have around 1000 genes are presented.

**Table 6:** Results of the study on noise sensitivity. Mean AUPR in the top 20 % of the possible connetions with a undirected evaluation with respect to intensity (*κ* *%*) of Gaussian local noise (*σ*_*Local*(*g*);*κ%*_). The best results are highlighted. Results obtained with current version (1.0) of the package and are updated online

Datasource	*κ*	ARACNE	C3NET	CLR	GeneNet	Genie3	MRNET	MutRank	MRNETB	PCIT	Zscore	Random
R1	0	0.008	0.004	0.010	0.140	0.025	0.010	0.040	0.010	**0.174**	0.133	0.001
	25	0.004	0.002	0.006	0.134	0.022	0.006	0.038	0.005	0.167	0.132	0.001
	50	0.002	0.001	0.003	0.121	0.020	0.003	0.031	0.003	0.150	0.130	0.001
	75	0.001	0.001	0.002	0.086	0.018	0.002	0.025	0.002	0.126	**0.127**	0.001
	100	0.001	0.001	0.001	0.006	0.015	0.001	0.017	0.001	0.097	**0.125**	0.001
S1	0	0.091	0.140	0.132	0.114	0.137	0.199	0.060	0.120	0.023	0.072	0.002
	25	0.040	0.033	**0.140**	0.059	0.133	0.112	0.062	0.126	0.059	0.035	0.002
	50	0.027	0.021	**0.139**	0.031	0.121	0.097	0.067	0.121	0.066	0.022	0.002
	75	0.021	0.014	**0.126**	0.024	0.104	0.076	0.066	0.098	0.063	0.011	0.003
	100	0.023	0.017	**0.119**	0.013	0.095	0.072	0.066	0.089	0.063	0.010	0.001
S2	0	0.014	0.039	0.046	0.025	0.044	0.060	0.021	0.039	0.008	0.001	0.001
	25	0.006	0.006	**0.044**	0.020	0.038	0.022	0.021	0.028	0.012	0.005	0.001
	50	0.004	0.003	0.044	0.016	0.033	0.016	0.020	0.026	0.012	0.002	0.001
	75	0.003	0.002	**0.040**	0.012	0.028	0.013	0.019	0.021	0.011	0.002	0.001
	100	0.003	0.002	**0.034**	0.008	0.022	0.011	0.017	0.015	0.010	0.001	0.001
G1	0	0.195	0.145	0.199	0.084	0.129	0.218	0.036	0.111	0.061	0.001	0.001
	25	0.113	0.107	**0.144**	0.082	0.111	0.142	0.035	0.086	0.060	<0.001	0.001
	50	0.069	0.065	**0.115**	0.074	0.091	0.096	0.031	0.073	0.057	0.001	0.001
	75	0.041	0.038	**0.082**	0.055	0.068	0.061	0.024	0.059	0.049	<0.001	0.001
	100	0.024	0.020	**0.048**	0.031	0.045	0.034	0.017	0.042	0.038	0.001	0.001
G2	0	0.163	0.147	0.131	0.038	0.077	0.177	0.025	0.062	0.045	0.002	0.001
	25	0.097	0.092	0.103	0.036	0.067	**0.124**	0.024	0.059	0.043	<0.001	0.001
	50	0.042	0.040	**0.079**	0.030	0.052	0.071	0.021	0.046	0.041	<0.001	<0.001
	75	0.019	0.018	**0.054**	0.019	0.038	0.038	0.016	0.034	0.034	<0.001	0.001
	100	0.011	0.009	**0.032**	0.008	0.025	0.021	0.011	0.026	0.025	<0.001	0.001

p < 0.05

### Sensitivity to number of experiments

The aim of this procedure is to measure the robustness of the different reconstruction methods in terms of number of available experiments. In a real world scenario, one has budgetary limitations and therefore there is a restriction on the number of different experiments that can be done. Here, we want to address this issue by identifying the best methods in several scenarios with different number of experiments. To do so, we subsample the experiments of the datasources of Table 2 with different number of experiments and then add local noise of 20 % of intensity. As in the noise sensitivity study, this process is repeated ten times and the performance metrics (*A* *U* *P* *R*_20 *%*_) are averaged over the different trials.

**Fig. 4:**
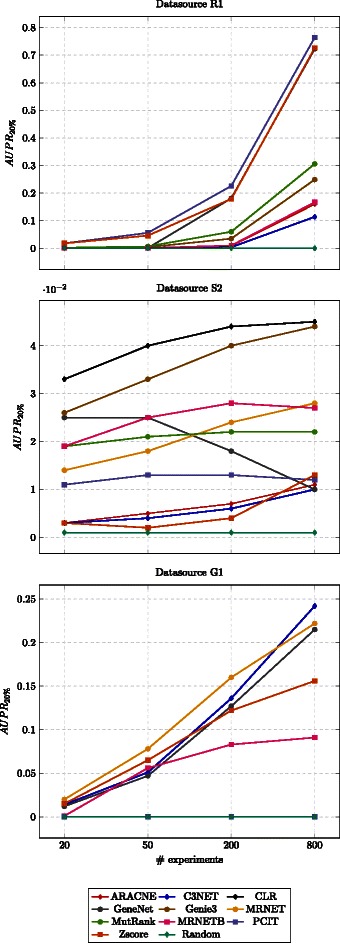
Plots of performance with different number of experiments. Each line represents a method (color coded), the mean performance over the ten runs is presented

The results are presented in Table 7. Figure 4 presents the results for one datasource of each simulator; to have a realistic setting we have chosen datasources that have more than 800 genes and one datasource for each simulator.

**Table 7:** Results of the study on the sensitivity with respect to the number of experiments. Mean AUPR in the top 20 % of the possible connetions with a undirected evaluation with respect to number of experiments (# exp). The best results are highlighted. Results obtained with current version (1.0) of the package and are updated online

Datasource	# exp	ARACNE	C3NET	CLR	GeneNet	Genie3	MRNET	MutRank	MRNETB	PCIT	Zscore	Random
R1	20	0.001	0.001	0.001	0.001	0.001	0.001	0.002	0.001	**0.018**	**0.018**	0.001
	50	0.001	0.001	0.001	0.001	0.004	0.001	0.007	0.001	0.056	0.046	<0.001
	200	0.008	0.004	0.010	0.181	0.035	0.010	0.060	0.010	0.226	0.179	0.001
	800	0.160	0.114	0.166	0.723	0.249	0.166	0.306	0.167	0.764	0.726	<0.001
S1	20	0.021	0.016	**0.113**	0.096	0.097	0.077	0.058	0.089	0.055	0.005	0.002
	50	0.027	0.020	**0.129**	0.099	0.122	0.091	0.060	0.110	0.057	0.017	0.002
	200	0.036	0.030	**0.138**	0.066	0.135	0.108	0.064	0.122	0.059	0.034	0.002
	800	0.064	0.054	0.141	0.053	**0.144**	0.139	0.065	0.130	0.059	0.058	0.003
S2	20	0.003	0.003	**0.033**	0.025	0.026	0.014	0.019	0.019	0.011	0.003	0.001
	50	0.005	0.004	**0.040**	0.025	0.033	0.018	0.021	0.025	0.013	0.002	0.001
	200	0.007	0.006	**0.044**	0.018	0.040	0.024	0.022	0.028	0.013	0.004	0.001
	800	0.011	0.010	**0.045**	0.010	**0.044**	0.028	0.022	0.027	0.012	0.013	0.001
G1	20	0.014	0.012	0.020	0.001	0.015	0.017	0.009	0.024	**0.029**	0.001	0.001
	50	0.051	0.047	**0.078**	0.056	0.065	0.064	0.020	0.063	0.048	<0.001	0.001
	200	0.136	0.127	**0.160**	0.083	0.122	0.164	0.038	0.090	0.061	0.001	0.001
	800	**0.242**	0.215	0.222	0.091	0.156	0.238	0.049	0.105	0.071	0.001	0.001
G2	20	0.012	0.010	0.022	0.001	0.012	0.017	0.007	**0.025**	**0.023**	0.001	0.001
	50	0.040	0.038	**0.064**	0.026	0.042	0.059	0.015	0.047	0.034	<0.001	0.001
	200	0.137	0.127	0.120	0.037	0.079	**0.157**	0.028	0.063	0.046	<0.001	0.001
	800	**0.246**	0.214	0.157	0.036	0.100	0.218	0.034	0.070	0.051	<0.001	0.001

p < 0.05

## Discussion

The results reveal that the studied methods exhibit different behavior across different simulators (and datasources), and none of the methods is the best one for all datasources. We also find large variations in terms of *A* *U* *P* *R*_20 *%*_ across datasources: Better results can be expected for smaller networks and for simpler simulators such as Rogers. It is worth noting that PCIT and Zscore almost reach a 100 % precision over their most confident connections in the Rogers datasets (see average precision-recall curves in supplemental material from Additional file 1: Figure S1, Additional file 2: Figure S2, Additional file 3: Figure S3, Additional file 4: Figure S4 and Additional file 5: Figure S5. This could be easily explained because both methods assume knockout experiments and normally distributed samples, in phase with how the data have been generated (by the Rogers simulator). As mentioned, none of the methods obtains the best results across the different datasources. But, as a general overview (see Fig. 2), we can observe that CLR is the best on the majority of the datasets. It is also one of the fastest methods in terms of computation time (see Table 3).

Differently from [5], we do not find the Zscore method as the best-performing method. However, there are several aspects to take into account. Our analysis evaluates only the most confident connections returned by the different methods whereas the study reported in [5] evaluates all the connections. The authors use the AUROC measure that could benefit the sparse recovered networks [15], as is the case of Zscore method. Furthermore, the analysis of [5] is based on simulation of the fully interventional data, knockouts and knockdowns, of the DREAM4 [12], and only involves the GNW simulator. Nevertheless, we also have evaluated the different reconstruction methods with the same setup as in [5] and also found that the Zscore is one of the best-performing methods when using knockout data.

### Effect of noise

We have studied the effect of noise on the performance using an additive Gaussian noise with different noise intensities, and we have found that the majority of the methods are quite robust to the noise effects. Also, the improvement of the performance on the datasets without noise is almost negligible. Even in the absence of noise, the *A* *U* *P* *R*_20 *%*_ values remain low, which highlights the difficulty of the task at hand. Still, we observe a trend of decreasing performance when the noise increases. However, we can see how the performances of ARACNE, C3NET and GeneNet are the most affected by increasing noises. The other methods appear less sensible to the noise addition.

### Effect of number of experiments

We also have studied the effect of the number of experiments on the performances. On one extreme, we have included a setup involving more experiments than genes and, on the other extreme, a setup where the number of experiments is around 1 % of the number of genes. We found that increasing the number of samples seems beneficial in most of the methods; it is worth noting that on datasource R1 the performance is outstanding for the Zscore, PCIT and GeneNet methods. These results are coherent with a similar study presented at [5]. Note that C3NET and ARACNE methods are the methods that suffer more the effects of a low number of experiments scenario. When few experiments are available the mutual information values between genes is more difficult to be estimated. The C3NET extracts the maximum value of MI per gene, while ARACNE eliminates the edge with minimum value of MI at every triangle.

## Review reproducibility

As previously stated, the present review is fully reproducible, with one function call of the Bioconductor package NetBenchmark. With this package, the different datasources are automatically loaded and the presented methods are implemented or imported with the package.

R is a broadly used open source language and environment for statistical computing and graphics [35]. Nowadays, it is a standard in statistical modeling, data analysis, bio-statistics and machine learning. There is a very active R community developing R packages implementing the latest advances in computational statistics. Moreover, platforms like Bioconductor host a huge amount of algorithms whose aim is the analysis and comprehension of genomic data mainly based on the R programming language [32]. Therefore, many GRN methods are implemented in an R package. This is why we chose to develop an R package to perform the benchmarking process in a fast and easy way.

We have developed several wrappers with the default parameters for most methods. The names of the wrappers of the GRN reconstruction algorithms that are currently available in the package are listed in Table 4. In order to reproduce the presented results, the user can run the commands provided in listing ?? after the download and installation of the package. Thanks to the seed of the random number generators of the different studies, the results are replicable.



In the present study we made a set of choices such as the evaluation measure or the number of datasets per datasource, but thanks to the Bioconductor package NetBenchmark, the user can make a different sets of choices, and the package can also be used for a deeper analysis of the methods. We refer the interested reader to the help files of the package for further information.

Additionally, the Bioconductor package NetBenchmark allows testing new methods with the benchmark in the same conditions that we presented in this review. The presented results are available online (https://imatge.upc.edu/netbenchmark/) that allows following research and comparison of new methods within the same conditions.

## Conclusions

In this paper, we have presented a new benchmark process for network reconstruction algorithms that relies on several in silico generators and a subsampling strategy to generate an environment for evaluating the different methods, in a fast and robust way. This benchmark is focused on (but not limited to) a GRN reconstruction task and therefore we have taken into account the goals of the community such as the evaluation of the most confident connections. We have also developed a Bioconductor package and webpage to allow future research and comparison of new methods under the same conditions and to provide the possibility to change them. The present paper has assessed the different GRN methods in a high-heterogeneity data scenario and has highlighted the specialization of methods for the different network types and data.

As a general conclusion, we can observe that CLR is the best on the majority of the datasets, but it does not obtain the best results across all the different datasources and kinds of data. In the case of complete knockout data, the best-performing methods are the Zscore followed by PCIT and GeneNet. Let us note also that Genie3 and MRNET exhibit competitive performances, however, these methods are not as fast as CLR in terms of computation time.

## Additional files

Additional file 1
**Figure S1.** Mean Precision Recall curves for the different GNR reconstruction methods at datasource R1. Each line is the mean curve over ten datasets. (EPS 242 kb)

Additional file 2
**Figure S2.** Mean Precision Recall curves for the different GNR reconstruction methods at datasource S1. Each line is the mean curve over ten datasets. (EPS 260 kb)

Additional file 3
**Figure S3.** Mean Precision Recall curves for the different GNR reconstruction methods at datasource S2. Each line is the mean curve over ten datasets. (EPS 266 kb)

Additional file 4
**Figure S4.** Mean Precision Recall curves for the different GNR reconstruction methods at datasource G1. Each line is the mean curve over ten datasets. (EPS 268 kb)

Additional file 5
**Figure S5.** Mean Precision Recall curves for the different GNR reconstruction methods at datasource G2. Each line is the mean curve over ten datasets. (EPS 268 kb)

## References

[1] Van den Bulcke T, Van Leemput K, Naudts B, van Remortel P, Ma H, Verschoren A (2006). Syntren: a generator of synthetic gene expression data for design and analysis of structure learning algorithms. BMC Bioinformatics.

[2] Altay G, Emmert-Streib F (2010). Revealing differences in gene network inference algorithms on the network level by ensemble methods. Bioinformatics.

[3] Schaffter T, Marbach D, Floreano D (2011). Genenetweaver: in silico benchmark generation and performance profiling of network inference methods. Bioinformatics.

[4] Marbach D, Costello JC, Küffner R, Vega NM, Prill RJ, Camacho DM (2012). Wisdom of crowds for robust gene network inference. Nat Methods.

[5] Maetschke SR, Madhamshettiwar PB, Davis MJ, Ragan MA (2014). Supervised, semi-supervised and unsupervised inference of gene regulatory networks. Briefings Bioinformatics.

[6] De Smet R, Marchal K (2010). Advantages and limitations of current network inference methods. Nat Rev Microbiol.

[7] Madhamshettiwar PB, Maetschke SR, Davis MJ, Reverter A, Ragan MA (2012). Gene regulatory network inference: evaluation and application to ovarian cancer allows the prioritization of drug targets. Genome Med.

[8] Roy S, Ernst J, Kharchenko PV, Kheradpour P, Negre N, Eaton ML (2010). Identification of functional elements and regulatory circuits by drosophila modencode. Science.

[9] Faith JJ, Hayete B, Thaden JT, Mogno I, Wierzbowski J, Cottarel G (2007). Large-scale mapping and validation of escherichia coli transcriptional regulation from a compendium of expression profiles. PLoS Biol.

[10] Rogers S, Girolami M (2005). A bayesian regression approach to the inference of regulatory networks from gene expression data. Bioinformatics.

[11] Olsen C, Meyer PE, Bontempi G (2009). On the impact of entropy estimation on transcriptional regulatory network inference based on mutual information. EURASIP J Bioinform Syst Biol.

[12] Marbach D, Prill RJ, Schaffter T, Mattiussi C, Floreano D, Stolovitzky G (2010). Revealing strengths and weaknesses of methods for gene network inference. Proc Natl Acad Sci.

[13] Stolovitzky G, Kundaje A, Held G, Duggar K, Haudenschild C (2005). Statistical analysis of mpss measurements: application to the study of lps-activated macrophage gene expression. Proc Natl Acad Sci U S A.

[14] Bansal M, Belcastro V, Ambesi-Impiombato A, di Bernardo D (2007). How to infer gene networks from expression profiles. Mol Syst Biol.

[15] Davis J, Goadrich M (2006). The relationship between precision-recall and roc curves. Proceedings of the 23rd International Conference on Machine Learning. ICML ’06.

[16] Marbach D, Roy S, Ay F, Meyer PE, Candeias R, Kahveci T (2012). Predictive regulatory models in drosophila melanogaster by integrative inference of transcriptional networks. Genome Res.

[17] Opgen-Rhein R, Strimmer K (2007). From correlation to causation networks: a simple approximate learning algorithm and its application to high-dimensional plant gene expression data. BMC Syst Biol.

[18] Obayashi T, Kinoshita K (2009). Rank of correlation coefficient as a comparable measure for biological significance of gene coexpression. DNA Res.

[19] Prill RJ, Marbach D, Saez-Rodriguez J, Sorger PK, Alexopoulos LG, Xue X (2010). Towards a rigorous assessment of systems biology models: the dream3 challenges. PloS ONE.

[20] Cover TM, Thomas JA (2006). Elements of Information Theory.

[21] Butte AJ, Kohane IS (2000). Mutual information relevance networks: functional genomic clustering using pairwise entropy measurements. Pac Symp Biocomput.

[22] Steuer R, Kurths J, Daub CO, Weise J, Selbig J (2002). The mutual information: detecting and evaluating dependencies between variables. Bioinformatics.

[23] Bellot P, Meyer PE. Efficient combination of pairwise feature networks. In: JMLR: Workshop and Conference Proceedings, Connectomics (ECML 2014). vol. 11, pp. 93–100 (2014).

[24] Margolin AA, Nemenman I, Basso K, Wiggins C, Stolovitzky G, Favera RD (2006). Aracne: an algorithm for the reconstruction of gene regulatory networks in a mammalian cellular context. BMC Bioinformatics.

[25] Reverter A, Chan EK (2008). Combining partial correlation and an information theory approach to the reversed engineering of gene co-expression networks. Bioinformatics.

[26] Altay G, Emmert-Streib F (2010). Inferring the conservative causal core of gene regulatory networks. BMC Syst Biol.

[27] Meyer PE, Kontos K, Lafitte F, Bontempi G (2007). Information-theoretic inference of large transcriptional regulatory networks. EURASIP J Bioinform Syst Biol.

[28] Ding C, Peng H (2005). Minimum redundancy feature selection from microarray gene expression data. J Bioinformatics Comput Biol.

[29] Meyer PE, Marbach D, Roy S, Kellis M. Information-theoretic inference of gene networks using backward elimination. In: BIOCOMP, International Conference on Bioinformatics and Computational Biology: 2010. p. 700–5.

[30] Huynh-Thu VA, Irrthum A, Wehenkel L, Geurts P (2010). Inferring regulatory networks from expression data using tree-based methods. PloS ONE.

[31] Breiman L (2001). Random forests. Mach Learn.

[32] Gentleman RC, Carey VJ, Bates DM, Bolstad B, Dettling M, Dudoit S (2004). Bioconductor: open software development for computational biology and bioinformatics. Genome Biol.

[33] Conover WJ, Conover W. Practical nonparametric statistics. 1980.

[34] Chambers JM (1983). Graphical Methods for Data Analysis.

[35] Ihaka R, Gentleman R (1996). R: a language for data analysis and graphics. J Computat Graph Stat.

